# Extracts of *Rhizoma Polygonati Odorati* Prevent High-Fat Diet-Induced Metabolic Disorders in C57BL/6 Mice

**DOI:** 10.1371/journal.pone.0081724

**Published:** 2013-11-29

**Authors:** Ming Gu, Yu Zhang, Shengjie Fan, Xiaobo Ding, Guang Ji, Cheng Huang

**Affiliations:** 1 School of Pharmacy, Shanghai University of Traditional Chinese Medicine, Shanghai, China; 2 College of Horticulture and Landscape Architecture, Southwest University, Chongqing, China; 3 Key Laboratory of Horticulture Science for Southern Mountainous Regions, Ministry of Education, Chongqing, China; 4 Institute of Digestive Disease, Longhua Hospital, Shanghai University of Traditional Chinese Medicine, Shanghai, China; Graduate School of Medicine, the University of Tokyo, Japan

## Abstract

*Polygonatum odoratum (Mill.) Druce* belongs to the *genus Polygonatum* family of plants. In traditional Chinese medicine, the root of *Polygonatum odoratum*, *Rhizoma Polygonati Odorati*, is used both for food and medicine to prevent and treat metabolic disorders such as hyperlipidemia, hyperglycemia, obesity and cardiovascular disease. However, there is no solid experimental evidence to support these applications, and the underlying mechanism is also needed to be elucidated. Here, we examined the effect of the extract of *Rhizoma Polygonati Odorati* (ER) on metabolic disorders in diet-induced C57BL/6 obese mice. In the preventive experiment, the ER blocked body weight gain, and lowered serum total cholesterol (TC), triglyceride (TG) and fasting blood glucose, improved glucose tolerance test (GTT) and insulin tolerance test (ITT), reduced the levels of serum insulin and leptin, and increased serum adiponectin levels in mice fed with a high-fat diet significantly. In the therapeutic study, we induced obesity in the mice and treated the obese mice with ER for two weeks. We found that ER treatments reduced serum TG and fasting blood glucose, and improved glucose tolerance in the mice. Gene expression analysis showed that ER increased the mRNA levels of peroxisome proliferator-activated receptors (PPAR) γ and α and their downstream target genes in mice livers, adipose tissues and HepG2 cells. Our data suggest that ER ameliorates metabolic disorders and enhances the mRNA expression of PPARs in obese C57BL/6 mice induced by high-fat diet.

## Introduction

Metabolic syndrome (MS) is a group of lipid and glucose metabolic disorders including obesity, hyperlipidemia, hyperglycemia and atherosclerosis etc [1], [2]. The increasing prevalence of MS has been considered as an epidemic public and economic problem worldwide [3]. Pharmacotherapy is the primary method of treating MS at present and prescription drugs dominate the main drug market for these diseases. Although clinical practices have repeatedly proven that prescription drugs are effective in the treatment of MS, side-effects such as liver and kidney toxicity cannot be ignored [4].

The use of functional food or dietary therapy for MS is attractive to the public. In China, many medicinal herbs such as coptis, ginseng, astragalus mongholicus and green tea are used in formulations for the prevention and treatment of MS and are safe and effective [5], [6]. Food-medicine dual plants that used as both food and medicine are important parts of traditional Chinese medicine. Several food-medicine duals, such as bitter melon, ginger, celery, citrus maxima, hawthorn and red kojic rice have been proven to be beneficial to the disorders of metabolism [7].

*Polygonatum odoratum (Mill.) Druce*, commonly known as Angular Solomon's-seal or Scented Solomon's-seal, belongs to the *genus Polygonatum*, which is widely distributed from Asia to Europe. *Rhizoma Polygonati Odorati*, the root of *Polygonatum odoratum*, is consumed as food in the East Asian countries [8]. In China, *Rhizoma Polygonati Odorati* is also used in herbal formulae to treat various diseases such as diabetes, hyperlipidemia, atherosclerosis and cancers. In addition, it has also been used for anti-oxidant purposes and increasing immune functions in traditional Chinese medicine [9]–[12].

Recently, the water extract of *Rhizoma Polygonati Odorati* has been shown to reduce blood glucose and to improve the glucose tolerance in diabetic mice and rats [13]. The saponin-rich fraction of *Polygonatum odoratum* has been shown to lower blood glucose in streptozotocin (STZ)-induced diabetic rats and to inhibit α-glycosidase activity [14]. A steroidal glycoside purified from *Polygonatum odoratum* has been reported to improve insulin resistance in 90% of pancreatectomized rats [9]. The total flavonoids of *Polygonatum odoratum* has also been shown to have anti-hyperglycemic effects in STZ and alloxan-induced diabetic rats [11]. However, little experimental data could support the effect of *Rhizoma Polygonati Odorati* on diet-induced metabolic disorders. Moreover, the underlying mechanism is still unknown.

Nuclear receptor transcription factor PPARs are important regulators of lipid and glucose hemostasis. PPARα is expressed highly in the liver, which is an organ involved in lipid metabolism. The activation of PPARα by its agonists could decrease serum TG levels and increase high density lipoprotein cholesterol (HDL-c) levels [15]–[17]. In contrast, PPARγ is present at higher concentrations in adipocytes, and PPARγ activation improves insulin sensitivity and reduces hyperglycemia [18]. Here, we show that the ethanol extract of *Rhizoma Polygonati Odorati* (ER) may prevent the development of hyperlipidemia and insulin resistance in high-fat diet-fed C57BL/6 mice and may increase PPARγ/α and their downstream genes.

## Materials and Methods

### Chemicals and Diet

*Rhizoma Polygonati Odorati* (Shanghai LeiYunShang Medicinal Materials Co.) weighed 500 g and was extracted using 75% ethanol for 4 hours at ethanol boiling point. The extract of *Rhizoma Polygonati Odorati* was concentrated at 40°C with a rotary evaporator under reduced pressure, freeze-dried to a powder, and dissolved in dimethylsulfoxide (DMSO) to the final concentration of 200 mg/ml for cell culture. Rosiglitazone (Ros) and WY14643 were purchased from Sigma-Aldrich (St. Louis, MO, USA). Ferulic acid and 5-hydroxymethylfurfural were obtained from the Shanghai Standard Product Center. High-fat diets (60% of calories derived from fat), and low-calorie diets (10% of calories derived from fat) were purchased from Research Diet (D12492, D12450B).

### Component Analysis in ER

The powder of ER was dissolved in water to the final concentration of 1% for component analysis. High Performance Liquid Chromatography (HPLC) analysis was performed on an Agilent 1200 liquid chromatograph system to determine the component in ER water solution. The compounds were monitored at 205 nm and 280 nm using a Discovery C18 HPLC Column (250×4.6 mm, 5 µm). The column was operated at 30°C, and the injection volume was 10 µL. The mobile phase consisted of 100% acetonitrile (A) and water containing 0.2% phosphoric acid (B) at a flow rate of 1.0 mL/min. The gradient profile was as follows: 0–10 min, 5–15% A; 10–45 min, 15–90% A; 45–50 min, 90–95% A, back to 5% A.

The content of total polysaccharide in ER water solution was determined using the phenol sulfate method [19]. The total flavonoid content of the methanolic extracts was measured using a colorimetric assay [20]. Total polyphenol content was measured using Folin-Ciocalteu (FC) reagent methods [21]. Residual protein was determined using Bradford protein assay measurement [22]. The quantity contents (%) of components in ER are shown in table 1.

**Table 1:** The quantity contents (%) of component in ER.

Component	Content (%)
Total polysaccharide	81.61%
Total flavonoid	0.26%
Total polyphenol	0.61%
Total amino acid	2.56%
The other components	14.94%

### Animals and Treatment

The animal protocols used in this study were approved by Shanghai University of Traditional Chinese Medicine (Approved Number 12003). Female C57BL/6 mice were purchased from the SLAC Laboratory (Shanghai, China). All animals were kept under controlled temperature (22–23°C) and on a 12-h light, 12-h dark cycle. For the preventive treatment, the six-week-old female C57BL/6 mice were randomly divided into three groups according to body weight: chow (10% of calories derived from fat), high-fat (HF, 60% of calories derived from fat), and high-fat plus 1% ER (ER was powered and mixed into HF diet at 1% (w/w) evenly). Mice were treated for 8 weeks. The food intake amount was measured by recording the food weight every 2 days through the experiment and the twenty-four-hour food intake amount was calculated.

For the therapeutic treatment, six-week-old mice were fed with a high-fat diet for 12 weeks to induce obesity. The obese animals were then randomly separated into either the HF or ER group, with the latter group being treated as the preventive treatment. The chow control mice continued to be fed the chow diet throughout the experiment. The mice were treated in this way for 2 weeks. Body weight and food consumption were recorded every 2 days.

### Rectal Temperature Measurement

The rectal temperature of the mouse was determined with a rectal probe attached to a digital thermometer (Physitemp, NJ, USA) according previous described method.

### Intraperitoneal Glucose Tolerance and Insulin Tolerance Test

At the end of the treatment, mice were fasted overnight (12 h). The baseline glucose values (0 min), prior to the injection of glucose (1 g/kg body weight), were measured by the means of collecting blood samples from the tail vein. Additional blood samples were collected at regular intervals (15, 30, 60, and 90 min) for glucose tolerance tests.

For intraperitoneal insulin tolerance test (IPITT), Non-fasted glucose levels were determined from the tail vein (0 min) from the mice. Then the insulin was injected intraperitoneally (0.75 U/kg body weight) (Sigma, St. Louis, MO). Subsequent blood samples were taken at 15, 30, 60, and 90 min after insulin administration for glucose measurement.

### Serum chemistry analysis

At the end of the animal experiment study, mice were anesthetized and cardiac blood was taken. Serum triglyceride (TG), total cholesterol (TC), HDL cholesterol (HDL-c), and LDL cholesterol (LDL-c) were measured using a Hitachi 7020 Automatic Analyzer (Hitachi, Ltd., Tokyo, Japan) with 100 µl of heart blood serum.

### Enzyme-Linked ImmunoSorbent Assay (ELISA)

Serum insulin, leptin and adiponectin levels were determined using enzyme-linked immunosorbent assay according the instruction from the manufactures. The mouse serum was stored at −80°C until analysis. The kits for insulin, leptin and adiponectin were purchased from ALPCO Diagnostics (Salem, NH, USA), R&D Systems (Oxon, UK) and AdipoGen (Seoul, Korea) respectively.

### Liver Lipid Content Analysis

The liver tissues were weighed and homogenized in lysis buffer (20 mM Tris-HCl pH 7.5, 150 mM NaCl, 1% Triton) and extracted with an equal volume of chloroform. The chloroform layers were dried and dissolved in isopropyl alcohol to measure lipid levels as described [23].

### Histological Analysis of Adipose

Adipose tissue was fixed in formalin, and paraffin-embedded section was prepared at 5 µm. The section was stained with hematoxylin and eosin according to a standard procedure.

### Quantitative Real-time PCR

Total RNA was extracted from the liver samples using spin columns (Qiagen, Germany) according to the manufacturer's instructions. Genomic DNA contamination was removed by using DNase I. The first-strand cDNA was synthesized with a cDNA synthesis kit (Fermentas, Madison, WI). An ABI StepOnePlus real-time PCR system (Applied Biosystems, USA) was used to analyze the gene expression levels. The cDNA was denatured at 95°C for 10 min followed by 40 cycles of PCR (95°C, 15 s; 60°C, 60 s). The primers used in the experiments were listed in Table 2. Beta -actin was used as an internal control to normalize all the mRNA levels.

**Table 2:** Sequences of the primers used in real-time PCR of the mouse tissue.

Gene	Forward primer	Reverse primer
β-Actin	TGTCCACCTTCCAGCAGATGT	AGCTCAGTAACAGTCCGCCTAGA
PPARγ	CGCTGATGCACTGCCTATGA	AGAGGTCCACAGAGCTGATTCC
PPARα	AGGCTGTAAGGGCTTCTTTCG	GGCATTTGTTCCGGTTCTTC
PPARβ	AGTGACCTGGCGCTCTTCAT	CGCAGAATGGTGTCCTGGAT
PGC-1α	TGTTCCCGATCACCATATTCC	GGTGTCTGTAGTGGCTTGATTC
PGC-1β	GGGTGCGCCTCCAAGTG	TCTACAGACAGAAGATGTTATGTGAACAC
ACC	GAATCTCCTGGTGACAATGCTTATT	GGTCTTGCTGAGTTGGGTTAGCT
ACO	CAGCACTGGTCTCCGTCATG	CTCCGGACTACCATCCAAGATG
aP2	CATGGCCAAGCCCAACAT	CGCCCAGTTTGAAGGAAATC
LPL	ATCGGAGAACTGCTCATGATGA	CGGATCCTCTCGATGACGAA
UCP-1	CATCACCACCCTGGCAAAA	AGCTGATTTGCCTCTGAATGC
UCP-2	GGGCACTGCAAGCATGTGTA	TCAGATTCCTGGGCAAGTCACT
UCP-3	TGGCCCAACATCACAAGAAA	TCCAGCAACTTCTCCTTGATGA
CD36	GCTTGCAACTGTCAGCACAT	GCCTTGCTGTAGCCAAGAAC
Glut4	GTAACTTCATTGTCGGCATGG	AGCTGAGATCTGGTCAAACG
TNFα	ATGGATCTCAAAGACAACCAACTAG	ACGGCAGAGAGGAGGTTGACTT
IL-1β	TCGTGCTGTCGGACCCATAT	GGTTCTCCTTGTACAAAGCTCATG
IL-6	AACCACGGCCTTCCCTACTT	TCTGTTGGGAGTGGTATCCTCTGT

HepG2 cells were seeded and grown in a 12-well plate to 80% confluence with high glucose DMEM containing 10% FBS at 37°C in 5% CO_2_, and then ER was added to the medium at 200 µg/mL for 24 hours. Total RNA was extracted, and genes expression analysis was performed as described above. The primers used in the cellular experiments were listed in Table 3.

**Table 3:** Sequences of the primers used in real-time PCR of HepG2 cell.

Gene	Forward primer	Reverse primer
β-Actin	AATCTGGCACCACACCTTCTA	ATAGCACAGCCTGGATAGCAAC
PPARγ	TGCTGTATTTGAATCCGACGTT	GCTCTTTAGAAACTCCCTTGTCATG
PPARα	ATCCCAGGCTTCGCAAACTT	CATGGCGAATATGGCCTCAT
aP2	GGGCCAGGAATTTGACGAA	GTACCAGGACACCCCCATCTAA
ACC	GGATGGTGTTCACTCGGTAATAGA	GGGTGATATGTGCTGCGTCAT
ACOX1	CCAAGCTTTCCTGCTCAGTGTT	CCCCCAGTCCCTTTTCTTCA
CD36	TGCTGTATTTGAATCCGACGTT	AAGGCCTTGGATGGAAGAACA
LPL	TATGCAGAAGCCCCGAGT	ATGAAGAGATGAATGGAG

### Transfection of Cultured Cells and Reporter Assays

The reporter assay was performed as previously described [24]. The expression plasmid pCMXGal-mPPARα, γ-LBD, and the Gal4 reporter vector MH100×4-TK-Luc were co-transfected with a reporter construct so that 1 µg of the relevant plasmid combined with 1 µg of reporter plasmids and 0.1 µg of pREP7 (Renilla luciferase) reporter to normalize transfection efficiencies. The transfection mixture, which contained 10 µg of total plasmids and 15 µl FuGENE-HD (Roche) per ml of DMEM, was added to HEK293T cells (ATCC) for 24 h and then removed. The PPARα and PPARγ agonists (WY14643 and Rosiglitazone), or ER (200 µg/ml), were added to fresh media and the cells were incubated for another 24 h to determine luciferase activity. We conducted the luciferase reporter assays using the Dual-Luciferase Reporter Assay System (Promega, USA), and the renilla luciferase activity was assayed to normalize transfection efficiencies. All of the transfection experiments were performed in triplicate and repeated at least three times independently.

### Statistical Analysis

Data analyses were performed using the statistical program SPSS 12.0 for Windows. All data were presented as means ± SE. Statistical analysis was performed using one-way analysis of variance (ANOVA). Differences were considered as significant (_*_), P<0.05 or not significant (NS), P>0.05.

## Results

### The composition in ER

**Figure 1:**
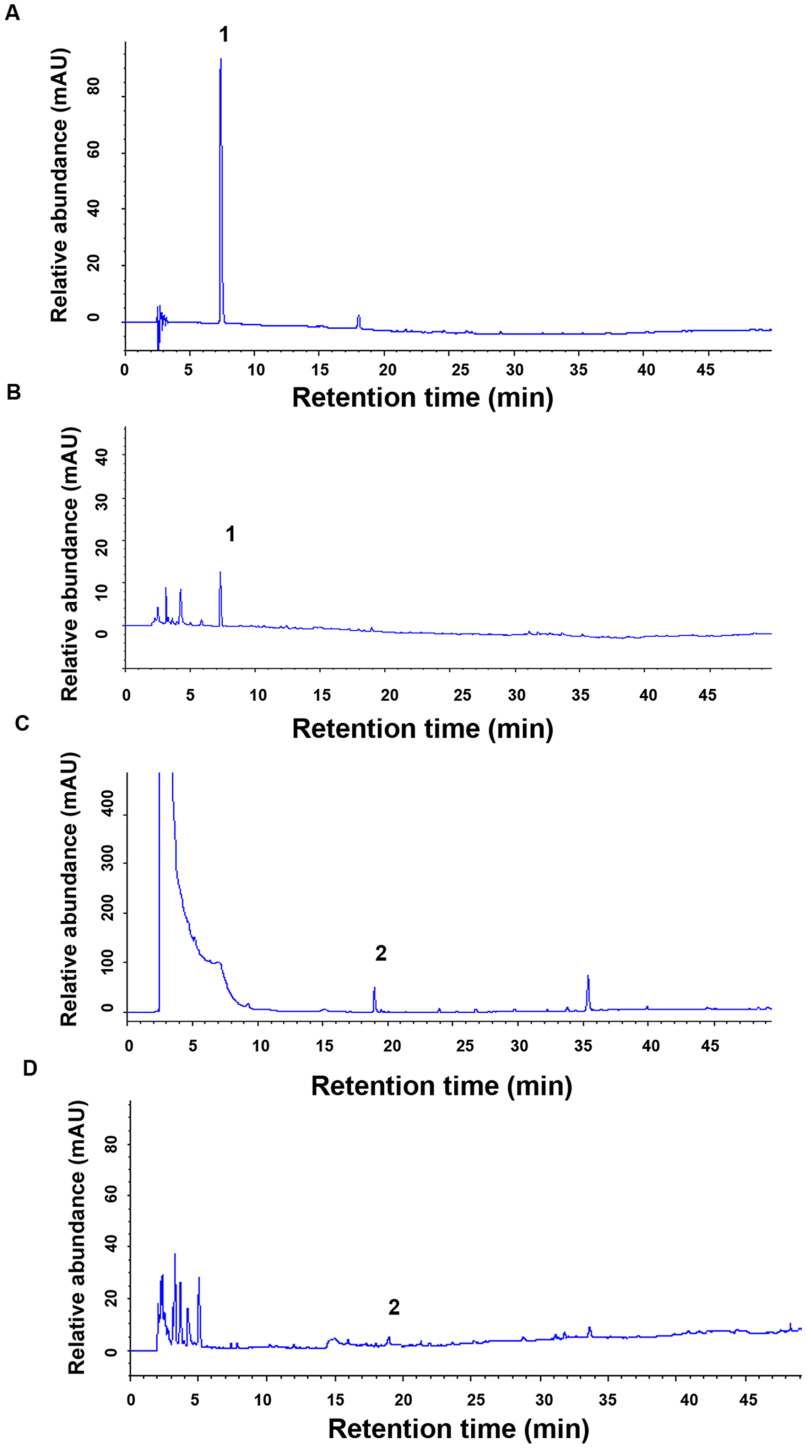
HPLC chromatograms of ER. (A) HPLC chromatogram of Standard substance of 5-hydroxymethylfurfural detected at 280 nm. (B) HPLC chromatogram of ER water solution detected at 280 nm. (C) HPLC chromatogram of Standard substance of ferulic acid detected at 205 nm. (D) HPLC chromatogram of ER water solution detected at 205 nm. (1) 5-hydroxymethylfurfural. (2) ferulic acid.

To determine the chromatographic profiles of ER, the extract was analyzed by HPLC at 205 nm (Figure 1A), and 280 nm (Figure 1B). A previous report has shown that *Rhizoma Polygonati Odorati* contains ferulic acid and 5-hydroxymethylfurfural [25]; therefore, these compounds were analyzed using HPLC at 205 nm and 280 nm, respectively, as a standard control (Figure 1A & C). Ferulic acid and 5-hydroxymethylfurfural were detected in the ER by comparing the retention of the chromatographic images of standards (Figure 1B and D). In addition, we found some large polarity compounds in ER.

To determine the main constituents in ER, we measured the contents of total polysaccharide, total flavonoid, total polyphenol and residual protein in ER. Our results showed that ER consists of 81.61% total polysaccharide, 0.26% total flavonoid, 0.61% total phenolic and 2.56% total amino acids (Table 1), indicating that polysaccharides are the main component of ER.

### ER prevents metabolic disorders in C57BL/6 mice induced by a high-fat diet

**Figure 2:**
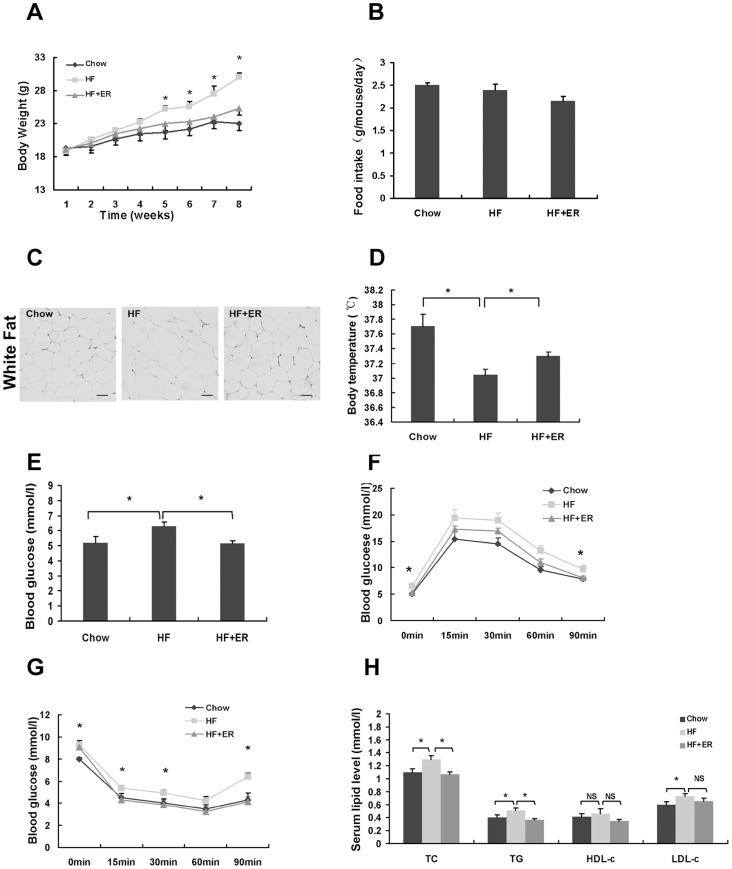
ER prevents metabolic disorders in C57BL/6 mice induced by high-fat diet. (A) Body weight. (B) Diet consumption. (C) Histological analysis of white adipose tissue (WAT). WAT was stained with hematoxylin and eosin. Scale bars represent 100 µm. (D) Body temperature. (E) Fasting glucose levels. (F) Glucose tolerance test. The mice were fasted for 12 h and the tail vein blood was used to measure the blood glucose levels. (G) Insulin tolerance test. (H) Serum TC, TG, HDL-c and LDL-c content. The data were showed as mean ± SEM. N = 7 for all groups. * P<0.05, NS: No significance.

To test whether ER blocks diet-induced metabolic disorders, C57BL/6 mice were fed with a high-fat (HF) diet or HF diet mixed with 1% ER for 8 weeks. The mice fed the HF diet displayed higher body weight gain compared to the Chow diet-fed mice after 8 weeks of treatment (Figure 2A). The ER-supplemented HF diet meanwhile, blocked the body weight gain of mice (Figure 2A, P<0.05). The food intake amount was not significantly different between the HF and ER groups (Figure 2B), indicating that the lower body weight in ER treated mice does not result from a lower calorie intake. Histological analysis showed the treatment of ER reduced the size of white adipocyte tissue (WAT) (Figure 2C), suggesting that ER could block adipocyte mass in HF diet-fed mice.

To test whether ER enhances energy expenditure, we measured body temperature, which is closely related to energy expenditure. The body temperature was remarkably increased after the treatment of ER (Figure 2D), suggesting that ER may increase energy expenditure in the obese mice which leads to reduction of energy storage in adipocyte.

Since ER blocks the body weight gain, we next investigated whether ER ameliorates fasting glucose, glucose intolerance and insulin resistance in vivo. Figure 2E showed that the fasting blood glucose level in HF-fed mice was markedly higher than that in chow control mice, while ER treatment significantly lowered the fasting glucose level in the mice (P<0.05). The HF-fed mice demonstrated impaired glucose and insulin intolerance compared with chow-fed mice, while the ER-treated group significantly improved fasting glucose level, glucose tolerance and insulin tolerance (Figure 2F–2G). We then assayed lipid levels of the mice. The HF-fed mice showed higher levels of serum TC, TG and LDL-c when compared to chow-control mice (Figure 2H, P<0.05). ER-treated mice had significantly reduced serum TC and TG levels compared to HF-control mice (Figure 2H, P<0.05). However, the LDL-c and HDL-c levels were not significantly altered (Figure 2H). These results suggest that ER could prevent the body weight gain and metabolic disorders induced by high-fat diet in mice.

### ER regulates serum insulin, adiponectin and leptin levels in high-fat diet-fed mice

**Figure 3:**
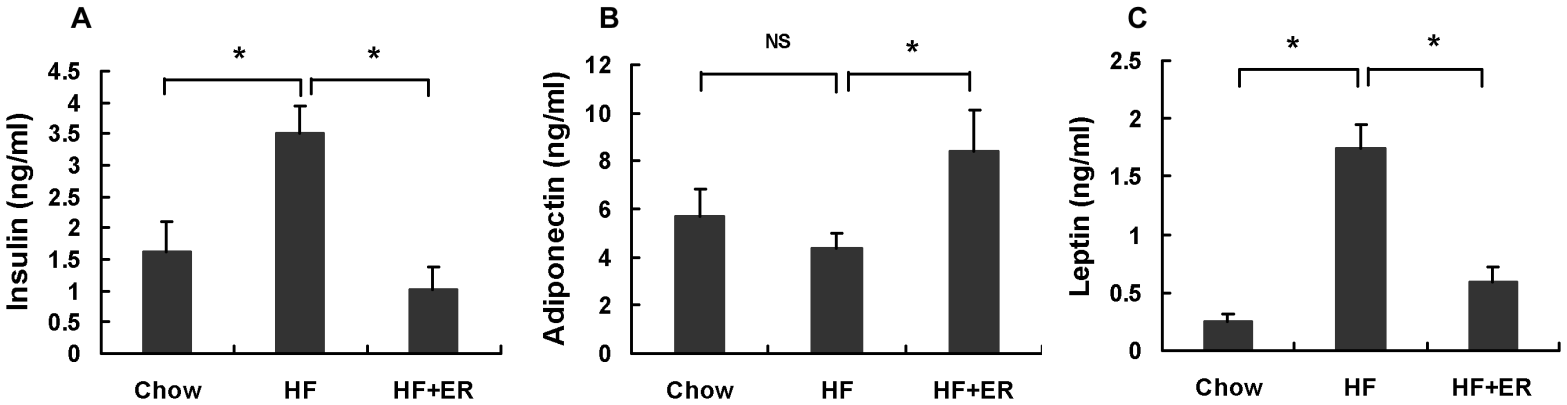
ER improves serum insulin, adiponectin and leptin levels in the obese mice. (A) Serum insulin. (B) Serum adiponectin. (C) Serum leptin. The data were showed as mean ± SEM. N = 7 for all groups. * P<0.05, NS: No significance.

Insulin resistance usually is companied by hyperinsulinemia in obese subjects which is associated with obesity, dyslipidemia, and glucose intolerance. To examine whether the insulin level was elevated in HF-fed mice, we tested the insulin contents using ELISA. The results showed that the insulin level was increased significantly in the HF-fed mice, while ER treatment markedly suppressed insulin level, indicating the ER could improve hyperinsulinemia in the mice (Figure 3A, P<0.05). Adiponectin, a adipocytokine, is key an important regulator of glucose metabolism. The combination of adiponectin and leptin has been reported to completely reverse insulin resistance in mice. ER increased serum adiponectin level in HF-diet-fed mice (Figure 3B, P<0.05). Similarly, leptin, the key regulator of body weight secreted by adipocyte, was significantly higher in HF-fed mice than that in chow-fed mice. ER reduced the leptin level of HF group notably (Figure 3C, P<0.05). Collectively, our data indicate that ER treatment could attenuate serum insulin and leptin levels and increase adiponectin in HF-diet induced obese mice which finally may be benefit to obesity and insulin resistance.

### ER lowers fasting glucose and TG levels in obese mice

**Figure 4:**
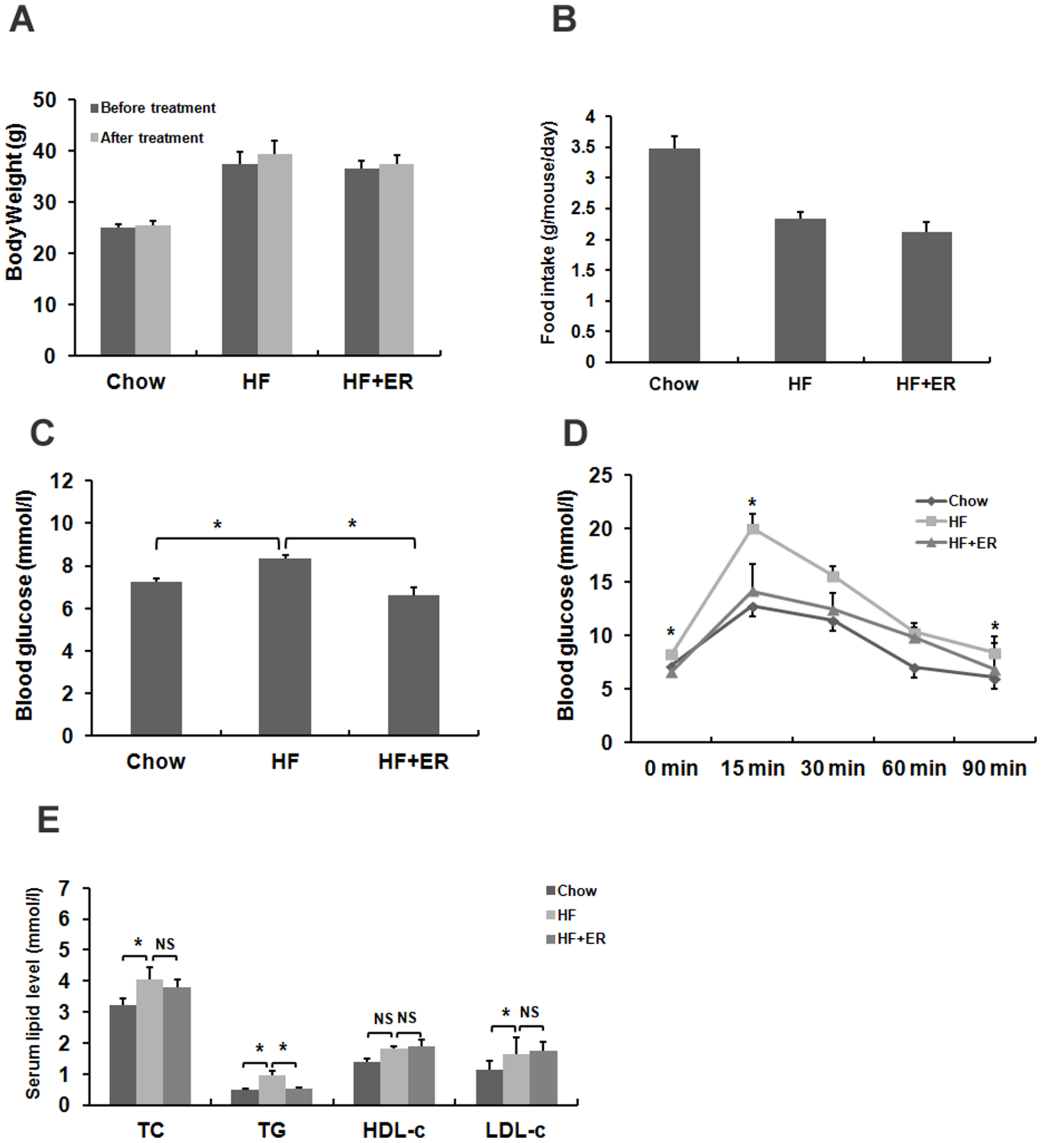
Therapeutic effect of ER on high-fat diet-induced obesity C57BL/6 mice. (A) Body weight gain. (B) Diet consumption. (C) Fasting glucose levels. The mice were fasted for 12 h and tail vein blood was used to measure the blood glucose levels. (D) Intraperitoneal glucose tolerance test (IPGTT). (E) Serum TC, TG, HDL-c and LDL-c content. The data were showed as mean ± SEM. N = 7 for all groups. * P<0.05, NS: No significance.

To study whether ER could alleviate metabolic disorders in obese mice, we fed the mice a HF diet for 12 weeks to induce obesity. The obese mice were then grouped and fed with HFD alone or HFD mixed with 1% ER for 2 weeks. Results showed that ER treatment did not notably reduce the body weight of the mice (Figure 4A). The food intake amount was not significantly different between the HF group and ER group (Figure 4B). To test whether ER could lower blood glucose level in DIO (Diet induced obesity) mice, we measured the fasting blood glucose levels and glucose tolerance in the mice. The HF-fed mice exhibited higher fasting blood glucose levels when compared to chow control mice, while the ER treated group showed lower glucose levels than HF-fed mice (Figure 4C, P<0.05). We then assayed intraperitoneal glucose tolerance and found that the ER significantly improved glucose tolerance at 15 min and 90 min following intraperitoneal injection of glucose in DIO mice (Figure 4D, P<0.05).

Next, we assayed the serum lipid levels of the mice. The HF-fed mice showed higher levels of serum TC, TG and LDL-c when compared to that in chow control mice (Figure 4E, P<0.05). ER treatment significantly lowered serum TG levels when compared to HF-feeding (Figure 4E, P<0.05). However, TC, HDL-c and LDL-c levels remained unchanged. Taken together, the results indicate that ER could improve glucose and lipid metabolism in the obese mice.

### ER improves lipid accumulation in the liver of C57BL/6 mice

**Figure 5:**
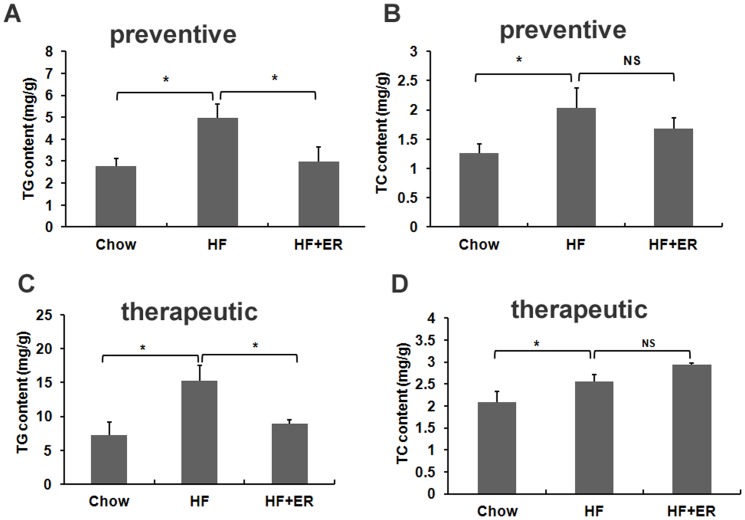
ER improves lipid accumulation in the liver of high-fat diet-induced. C57BL/6 mice. TG (A) and TC (B) contents in the mouse liver of preventive treatment. TG (C) and TC (D) contents in the mouse liver of therapeutic treatment. The data were shown as means ± SEM. n = 7 for all groups. * P<0.05, NS: No significance.

Next, we measured TG and TC contents in the mouse livers of the preventive and therapeutic experiments. In the preventive treatment, TG and TC levels in the HF diet-fed mice were markedly increased when compared to those in the chow diet control mice, whereas ER treatment significantly decreased TG accumulation in the livers of the HF diet-fed mice (Figure 5A, P<0.05), but did not change the TC levels (Figure 5B). In therapeutic treatment, the TG and TC levels in the obese mouse liver were markedly higher than those in the control mouse liver, similarly ER treatment also significantly lowered TG contents in the obese mouse liver (Figure 5C, P<0.05). However, TC content in ER-treated mouse liver was not significantly changed compared to that in obese control mice, indicating that the ER could improve TG accumulation in mouse livers (Figure 5D).

### ER up-regulates genes expression levels of PPARs

**Figure 6:**
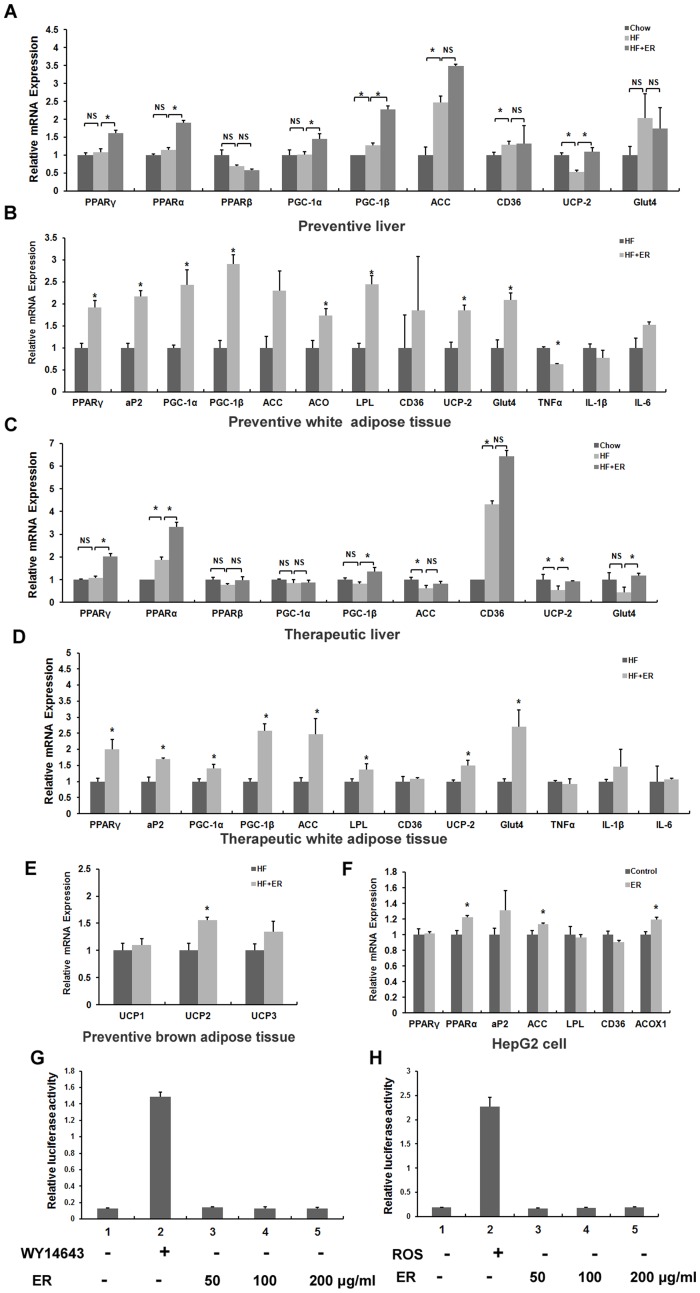
ER regulates genes related to glucose and lipid metabolism in PPAR pathway. The relative gene expression levels in the livers (A) and white adipose tissues (B) from ER-treated and HF-fed mice in the preventive treatment. The relative gene expression levels in the livers (C) and white adipose tissues (D) from ER-treated and HF-fed mice in the therapeutic treatment. (E) The relative gene expression levels in the brown adipose tissues from ER-treated and HF-fed mice in the preventive treatment. (F) The relative gene expression levels in ER-treated and control HepG2 cells. Beta-actin was used as an internal control for normalizing the mRNA levels. Data are presented as means ± SE for seven mice per group or six repeat cell samples per group, * P<0.05 versus HF group or HepG2 control group, NS: No significance. (G–H) ER cannot regulate PPARα and γ transcription activity. GAL4-DBD-LBD expression plasmids and a GAL4-responsive luciferase reporter were co-transfected into HEK293T cells for 24 h and treated with the PPARα and PPARγ agonists WY14643 and rosiglitazone (20 µM), and ER (50, 100, and 200 µg/ml) for another 24 h. The relative luciferase activities were measured by comparison to renilla luciferase activities. The results represent three independent experiments, and data are presented as means ± SEM.

The expression of PPARs and their downstream target genes is important for the regulation of lipid and glucose homeostasis. We evaluated the effects of ER on the expression of PPARs and their target genes by analyzing the mRNA expression levels in the livers and white adipose tissues from chow control, HF-fed control and ER treated mice in preventive and therapeutic treatments. Our results showed that HFD feeding increased the mRNA levels of PPARα, peroxisome proliferator-activated receptor gamma coactivator 1 (PGC1-β), and cluster of Differentiation 36 (CD36) and decreased uncoupling protein 2 (UCP-2) mRNA expression in the mouse livers from preventive and therapeutic treatments compared with chow diet feeding (Figure 6A and C, P<0.05). In preventive treatment, the mRNA levels of PPARα, γ, PGC1-α, β, and PPAR target genes, UCP-2 and glucose transporter 4 (Glut4), were up-regulated significantly in livers of mice treated by ER compared with those in livers of mice fed HFD (Figure 6A, P<0.05). Whereas the mRNA levels of PPARγ, PGC1-α, β, adipose fatty acid-binding protein 2 (aP2), lipoprotein lipase (LPL), acyl-CoA oxidase (ACO), UCP-2 and Glut4 in white adipose tissues were also increased by ER treatment (Figure 6B, P<0.05).

In therapeutic treatment, ER treatment significantly increased the mRNA expression of PPARγ, α, PGC1-β, and UCP-2 and Glut4, in the livers of mice (Figure 6C, P<0.05). The mRNA expression levels of PPARγ, PGC1-α, PGC1-β, aP2, LPL, acetyl coenzyme A carboxylase (ACC), UCP-2 and Glut4 were also increased in the white adipose tissues of ER treated mice compared with those in livers and white adipose tissues from HFD control mice (Figure 6D, P<0.05).

We also conducted genes expression analysis of thermogenic markers UCPs in brown adipose tissues and inflammatory cytokines tumor necrosis factor α (TNFα), interleukin 1β (IL-1β) and IL-6 in white adipose tissues from ER treated and HF-fed control mice. The results showed that ER increased the mRNA expression of UCP-2 (Figure 6E, P<0.05), but not UCP-1 and -3 in brown adipose tissues, and decreased the mRNA expression of TNFα, but not IL-1β and IL-6 in white adipose tissues in preventive treatment mice (Figure 6B, P<0.05), suggesting ER may regulate thermogenesis and inflammation.

We then analyzed the gene expression level in ER-treated HepG2 cells. The result showed that 24 hour treatment of ER significantly enhanced the expression of PPARα and its target genes ACOX1 and ACC in HepG2 cells (Figure 6F, P<0.05). Collectively, these results indicate that ER may enhance genes expression of PPARs and their target genes both in vivo and in vitro.

### ER has no influence on PPARα and γ transcription activities

Nuclear receptor transcription factors are important regulators of lipid and glucose homeostasis through the modulation of the expression of downstream target genes. To analyze whether ER could activate the transcription activities of nuclear receptor transcription factors, we determined PPARα, and -γ transcription activities with a reporter assay system. The results showed that ER did not change PPARα or -γ transcription activity (Figure 6G and H), indicating that ER does not contain the ligands of PPAR α and -γ.

## Discussion

*Rhizoma Polygonati Odorati* is used as both food and medicine for lipid and glucose metabolic disorders in the East Asian countries. Although it has been used in clinics for more than 2000 years, little experimental data has proven its effect or shown its pharmacological mechanism. In the present study, we provided evidence that ER can prevent metabolic disorders and especially ameliorate dyslipidemia in high-fat diet-fed mice. Our results showed that ER could prevent and treat the metabolic disorders in the mice fed with high-fat diets.

Polysaccharides have emerged as an important source of biological activities including antioxidant, immunomodulation, anti-inflammation, antitumor, neuroprotection, radioprotection, anti-diabetes, hepatoprotection, anti-osteoporosis and anti-fatigue properties [26]–[28]. Recent studies have shown that polysaccharides purified from various plants attenuated hyperglycemia in diabetic animals [29]–[34]. We found that the ER contains up to 81.61% of polysaccharides. Thus, it is reasonable to propose that polysaccharides may be one of the ingredients responsible for those glucose lowering activities of the ER.

In the present study, the preventive treatment of the ER blocked the body weight gain induced by a HF diet, while the food intake was not changed markedly, indicating that the effect is not caused by a decrease of calorie intake. However, in the therapeutic experiment, the body weight of the obese mice was not decreased, which indicated that ER could prevent body weight gain but could not reduce the body weight of obese subjects. Our results also showed that both preventive and therapeutic treatments lowered blood glucose, serum TG and liver TG contents in the mice fed HF diets, suggesting that ER could effectively lower HF diet-induced hyperglycemia and hyperlipidemia. However, ER treatment attenuated TC contents in preventive treated mice, but not in obese mice. This discrepancy may be caused by the shorter term of therapeutic treatment and the different conditions in the mice. In the preventive therapy, we treated mice for 8 weeks, but only 2 weeks was used for therapeutic treatment. Extension of the therapeutic time may achieve better results.

Hyperinsulinemia and hyperleptinemia indicates the insulin and leptin resistance, which is associated with obesity, dyslipidemia, and glucose intolerance [35], [36]. We induced the leptin and insulin resistance in the mice with 8 weeks high-fat diet feeding, which similar to the previous report [37], [38]. ER treatment significantly lowered the circulating insulin and leptin in the mice. Token together with finding of GTT and ITT, the dada indicate that ER could ameliorate leptin and insulin resistance in the mice. The increases of circulating adiponectin may improve insulin sensitivity in obese and diabetes patients [39]. Although HF diet did not reduce the concentration of adiponectin, ER increased the serum adiponectin significantly, which may be benefit to the insulin resistance and obesity.

Peroxisome-proliferator-activated receptors (PPARs) are nuclear receptor transcription factors that have been identified as drug targets for metabolic disorders. These receptors are lipid and glucose metabolism sensors and exist in adipocytes, livers and muscle, etc. [18], [40]. Previous studies have shown that activators of PPARs can ameliorate glucose and lipid metabolic disorders by modulating genes related to lipid and glucose metabolism [41], [42]. TZDs have been shown to lower blood glucose via the activation of PPARγ signaling, while fibrates attenuate serum TG as PPARα agonists [43]–[45]. Recently, many herbal or natural products have been reported as activators or modulators of PPARs [46], which are beneficial for metabolic disorders or reduce the side-effects of current therapies [47], [48]. For example, Orsolya *et al*. reported that soy isoflavones exert a beneficial hypolipidemic and anti-diabetic effect through the activation of PPARs [49]. Serisier *et al*. reported that green tea increases insulin sensitivity, decreases plasma TG concentrations and increases the expression of PPARα, PPARγ and their target genes LPL, Glut4 and adiponectin [50]. Mi-Young *et al*. found that Korean red ginseng and banaba leaf water extracts have an effect on glucose homeostasis via the up-regulation of PPARs [51].

Our data showed that ER significantly increased the genes expression of PPARα and -γ in the liver, adipocyte tissues and HepG2 cell, but did not enhance the transcription activities of these receptors. Currently, only the agonists of PPARs have been used in the treatment of these diseases. However, recent studies have shown that the increase mRNA expression of PPARα and -γ also have significant therapeutic influences on the metabolic disorders [47], [52], [53]. Therefore, we proposed that ER may alleviate the metabolic conditions via the enhancement of PPAR signaling.

PPARs participate in mediating metabolic disorders via downstream genes which are important for adipocyte maturation, lipid accumulation, and insulin-sensitive glucose transport, including PGC1-α, PGC1-β, aP2, LPL, ACC, ACO, CD36, UCP-2 and Glut4 [18], [42], [54]. PGC1-α and PGC1-β are co-activators of PPARγ, which increases lipogenesis and lipoprotein transport in the liver [55]. As a typical target gene, aP2 modulates inflammatory responses and cholesterol ester accumulation [56]. LPL has effect on decomposition of lipoprotein triglyceride of nuclear. ACC and ACO are involved in triglyceride synthesis, the expression levels of which are known to increase in the steatotic livers of ob/ob mice [57]. CD36 is recognized as a lipid and fatty acid receptor and plays an important role in metabolic syndrome and associated cardiac events [58], [59]. UCP-2 may act as an important regulator of energy and lipid metabolism, insulin resistance, glucose utilization, and regulation of reactive oxygen species [60], [61]. Increasing the expression of UCP-2 may help to prevent the development of hepatic steatosis and steatohepatitis atherosclerosis and obesity [62]. Glut4, the major insulin-regulated glucose transporter, is mainly expressed in skeletal and cardiac muscle and adipocytes; adipose-specific Glut4–KO (AG4KO) mice have showed fasting hyperglycemia [63], [64]. Therefore, we tested the gene expression of PPARs and its downstream targets in livers and white adipose tissues from HF-fed mice and ER-treated mice and in HepG2 cells. We found that ER up-regulated the gene expression of PPARs and its targets. Our data indicate that ER may regulate lipid and glucose disorders via the induction the expression of PPARs. These data further support the modulation of PPAR signaling by ER.

Our data showed ER blocked the body weight gain induced by HF diet in preventive study. Adipocyte differentiation is along with the increasing expression of inflammatory cytokines [65]. So we tested the inflammatory cytokines expression in white adipose tissues, the result showed that ER treatment decreased the expression of TNFα, but not IL-1βand IL-6in white adipose tissues from preventive treatment. Body weight change is associated with energy expenditure. We found ER markedly increased body temperature of the mice. To confirm that ER could enhance energy metabolism, we then assayed the expression of UCPs, the thermogenic markers, in brown adipose tissues from HF-fed mice and ER-treated mice in preventive study. We found that ER enhanced UCP-2 mRNA expression, whereas UCP-1 and -3 remained no significant change. Although the biological function of UCP2 is still open to dispute [66], it has been identified as a critical regulator of cellular fuel utilization and whole body glucose and lipid metabolism recently [67]. Thus we postulated that ER may increase energy expenditure partly through the increase of UCP-2 expression in the mice.

In summary, our results provided evidence that the ER plays a role in ameliorating metabolic disorders in high-fat diet-fed mice. ER also up-regulated the mRNA expression of PPARγ, α and their target genes in the mice. ER may be a choice as a safe dietary strategy for preventing metabolic disorders. Further investigation is needed to define the mechanisms by which each component protects against metabolic disorders and whether other mechanisms exist or not.
